# Competing Nonadiabatic Relaxation Pathways for Near-UV Excited *ortho*-Nitrophenol in Aqueous Solution

**DOI:** 10.1021/acs.jpclett.4c02154

**Published:** 2024-08-29

**Authors:** Hallam J. M. Greene, Deborin Ghosh, Igor V. Sazanovich, Ryan Phelps, Basile F. E. Curchod, Andrew J. Orr-Ewing

**Affiliations:** † School of Chemistry, University of Bristol, Cantock’s Close, Bristol BS8 1TS, U.K.; ‡ Central Laser Facility, Research Complex at Harwell, Science and Technology Facilities Council, Rutherford Appleton Laboratory, Harwell Oxford, Didcot, Oxfordshire OX11 0QX, U.K.

## Abstract

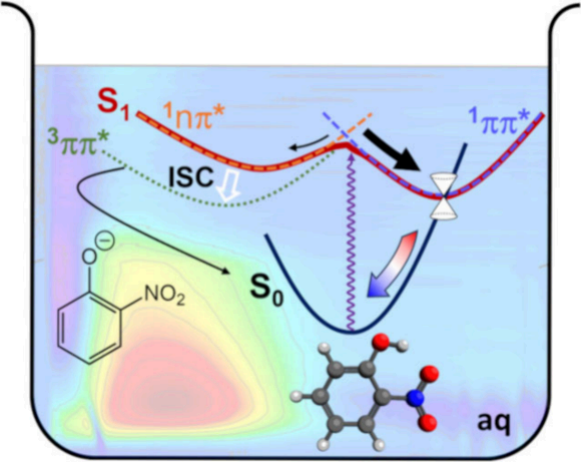

Nitrophenols are atmospheric pollutants found in brown carbon aerosols produced by biomass burning. Absorption of solar radiation by these nitrophenols contributes to atmospheric radiative forcing, but quantifying this climate impact requires better understanding of their photochemical pathways. Here, the photochemistry of near-UV (λ = 350 nm) excited *ortho*-nitrophenol in aqueous solution is investigated using transient absorption spectroscopy and time-resolved infrared spectroscopy over the fs to μs time scale to characterize the excited states, intermediates, and photoproducts. Interpretation of the transient spectroscopy data is supported by quantum chemical calculations using linear-response time-dependent density functional theory (LR-TDDFT). Our results indicate efficient nonradiative decay via an S_1_(ππ*)/S_0_ conical intersection leading to hot ground state *ortho*-nitrophenol which vibrationally cools in solution. A previously unreported minor pathway involves intersystem crossing near an S_1_(nπ*) minimum, with decay of the resulting triplet *ortho*-nitrophenol facilitated by deprotonation. These efficient relaxation pathways account for the low quantum yields of photodegradation.

Brown carbon (BrC) aerosols composed of organic particles dispersed in air absorb sunlight in the UV and visible regions at wavelengths >300 nm and thus have a significant impact on radiative forcing in the lower atmosphere.^1,2^ Nitroaromatic compounds are a major component of BrC, accounting for about half of the light absorption by these aerosols.^2^ A major class of nitroaromatic pollutants, nitrophenols are formed from biomass burning, alongside emissions from vehicles and release from industrial sources.^2−5^ Nitrophenols may also form as a result of secondary reactions between aromatic molecules, OH radicals and nitrogen oxides in the atmosphere.^1,5^ As well as contributing to BrC, *ortho*-substituted nitrophenols, such as *ortho*-nitrophenol (oNP - Figure 1a) have been identified as a source of nitrous acid (HONO) when photoexcited in the gas phase, albeit at low quantum yields.^6,7^

**Figure 1:**
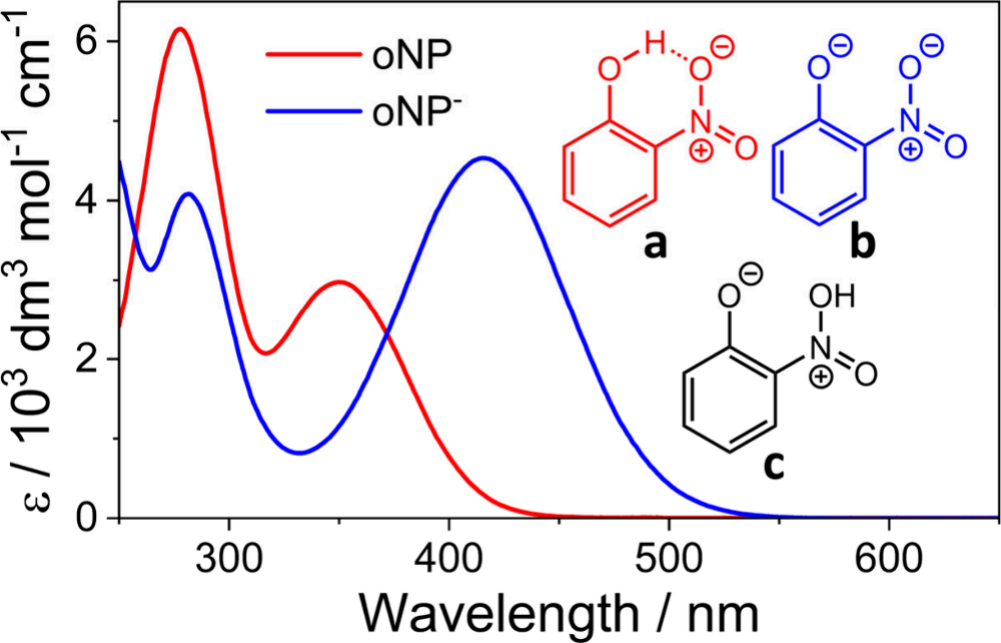
UV/vis absorption spectra of *ortho*-nitrophenol (oNP) and *ortho*-nitrophenolate (oNP^–^) in aqueous solution with inset structures: (a) *ortho*-nitrophenol in its nitro form, showing the intramolecular H-bond; (b) *ortho*-nitrophenolate, the anion formed by deprotonation; (c) the *aci*-nitro tautomer following proton transfer from the OH to the nitro group.

Studying the photochemical behavior of oNP in aqueous solution is of importance in understanding how it may behave in aqueous atmospheric aerosols, cloudwater and fog droplets exposed to sunlight. These droplets exhibit a wide range of pH,^8^ and thus the pH-dependence of the photochemistry should also be considered. oNP has a p*K*_a_ of 7.2,^9,10^ and thus the dynamics of the anion (oNP^–^ - Figure 1b) could influence the photochemistry of this species in solutions of higher pH. Both the neutral and anionic species have low quantum yields for photodegradation in aqueous solution (Φ = 5 × 10^–6^ and 2 × 10^–6^ respectively for irradiation at 365 nm),^11^ and no fluorescence or phosphorescence emission has been reported for oNP or its anion, indicating efficient nonradiative decay pathways. The ultrafast dynamics of photoexcited oNP in aqueous solution have previously been studied by Ernst et al. by femtosecond transient absorption (TA) spectroscopy, pumping at a wavelength of 350 nm and using 15 discrete probe wavelengths in the range 480–1100 nm, with time delays of up to 500 ps.^12^ The dynamics of the photoexcited anion were described by Michenfelder et al.^13^ and Bailey-Darland et al.^10^ Previous computational studies have also investigated the UV-induced dynamics of oNP in the gas and aqueous phase.^14,15^

In this work, TA spectroscopy using time delays seamlessly spanning femtosecond-to-microsecond time scales is used to investigate both the early and later time photochemistry of oNP, with white light continuum probes employed to better understand the evolution of the transient spectra. Time-resolved infrared spectroscopy further characterizes the states and species involved and the degree of parent-molecule recovery. Quantum chemical calculations using linear-response time-dependent density functional theory (LR-TDDFT), benchmarked with ADC(2), provide a framework for the interpretation of the experimental results. From these measurements and calculations, a comprehensive picture emerges for the relaxation pathways of UV-photoexcited aqueous oNP and their associated time scales.

The TA spectra of oNP in aqueous solution presented in Figure 2 show four distinct features when pumped into the first bright state at its absorption maximum at 350 nm (see Figure 1). Band assignments are provided in SI Figure S1. A positive feature with a maximum at around 450 nm (i) is assigned to excited state absorption (ESA) from the singlet ππ* state. A negative feature in the range 600–700 nm (iii) is attributed to stimulated emission from the same state. A blue-shifting peak in the range 400–450 nm (ii), which overlaps with peak (i) in early time, is attributed to hot ground state absorption (HGSA) from vibrationally excited oNP in its S_0_ state.

**Figure 2:**
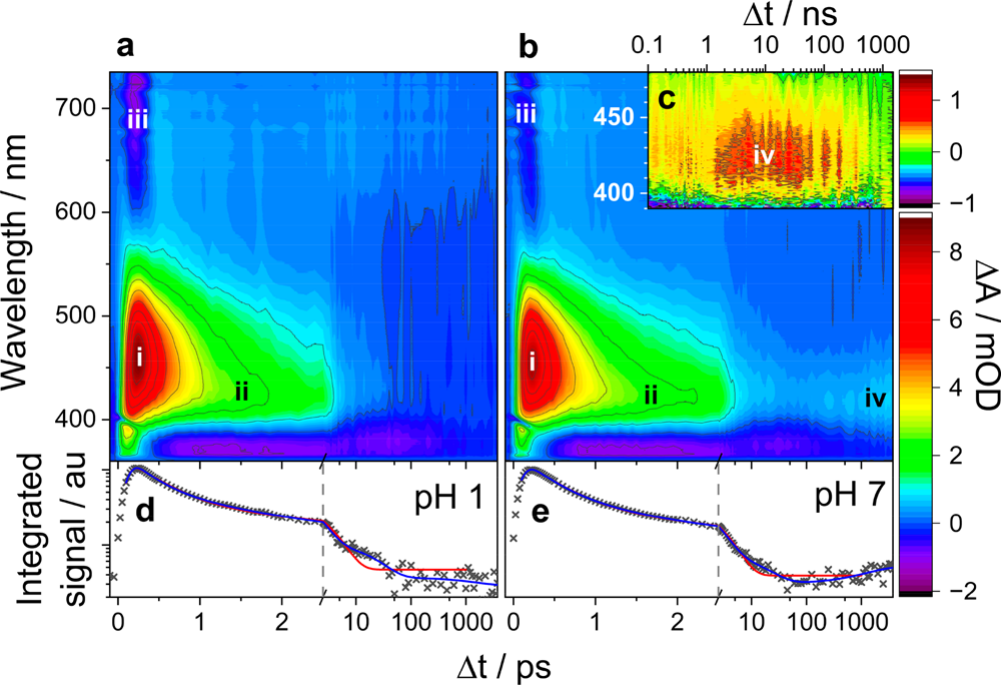
Transient absorption spectra for *ortho*-nitrophenol photoexcited at 350 nm in aqueous solution (a) with 0.1 M HCl, and (b) at its intrinsic pH shown as contour plots; (c) Contour plot of the 100 ps −1 μs transient absorption spectra of oNP in aqueous solutions at its intrinsic pH, measured using electro-optical delays; (**d**,**e**) Kinetic traces of an integrated region from 400–600 nm, from the data shown in (a) and (b) respectively, fitted to a biexponential function (red) or higher order exponential (blue), both with a Gaussian convolution to account for the IRF; time constants are given in Table 1. The time delay Δt is plotted on a split linear-logarithmic *y* axis in (a,b,d,e) and a logarithmic axis in (c). Highlighted features show i – excited state absorption (ESA) from the first bright state, ii – hot ground state absorption (HGSA), iii – stimulated emission (SE) from the first bright state, iv – absorption from the deprotonated anion.

Data were collected for a solution at its intrinsic pH – denoted pH 7–and a solution containing 0.1 M HCl–denoted pH 1 (both values omit contributions from the weakly acidic oNP). A long-lived feature (iv) is visible only in late time in the spectra collected at pH 7. Further details of the behavior of iv can be seen in Figure 2c which shows the TA spectra measured for a similar solution using electro-optic delays (EOD) to extend the range of Δt beyond 1 μs. The peak has a center around 420 nm which matches that of the deprotonated oNP in its ground state (Figure 1), and this species does not accumulate in solution of low pH, thus the peak is attributed to the *ortho*-nitrophenolate anion (oNP^–^ - Figure 1b). This assignment is supported by TRIR spectra reported below.

The delayed onset of absorption by oNP^–^ indicates that it forms from the triplet state. The growth of the oNP^–^ peak is multiple orders of magnitude slower than the decay of the first bright state, so deprotonation from this singlet state of oNP to form the S_0_ anion can be discounted. Deprotonation from an excited singlet state of oNP instead producing the S_1_ state of the anion can also be discounted because of the known sub-ps electronic deexcitation of the oNP^–^ S_1_ state.^10,13^ The photoacid behavior of the triplet state is discussed in more detail below.

To investigate the kinetics, the overlapping ESA and HGSA features in early time TA spectra were fitted to a biexponential decay with a Gaussian convolution to account for the instrument response. The resulting time constants are reported in Table 1. The ∼400 fs time constant τ_1_ can be attributed to depopulation of the ππ* excited electronic state, and the ∼3 ps τ_2_ to vibrational cooling of the resulting internally hot molecules in the S_0_ state. The kinetics of these positive TA features in early time spectra are similar to those determined by Ernst et al., although the ∼3 ps component is here attributed to vibrational cooling of the hot ground state, rather than to decay of the singlet state by intersystem crossing (ISC).^12^ This HGSA assignment is confirmed by our TRIR measurements (see below). An alternative fitting using a higher-order exponential gives similar values for the depopulation of the ππ* excited electronic state τ_1_, and slightly faster hot ground state cooling τ_2_ on the order of 2 ps, which is similar to the 1.4 ± 0.3 ps measured for hot ground state cooling in the anion.^13^ This fitting also gives a weakly contributing third component, τ_3_, attributed to depopulation of S_1_ by ISC, which is an order of magnitude slower. As we show later, branching into the triplet states of oNP is only a minor pathway for relaxation.

**Table 1:** Time Constants Derived from Integrated Regions of the TA Data^a^

Data set		pH 7	pH 1		pH 7 EOD
	Integration region (nm)	400–600 nm	400–600 nm		400–475 nm
	Time window fitted	0.1–1000 ps	0.1–1000 ps		0.1–4000 ns
Biexponential Fitting	τ_1_	410 ± 10 fs	400 ± 20 fs	τ_4_	1.5 ± 0.5 ns
	τ_2_	3.0 ± 0.1 ps	3.4 ± 0.2 ps	τ_5_	390 ± 50 ns

	Time window fitted	0.1–3750 ps	0.1–3750 ps		
Higher-Order Fitting	τ_1_	380 ± 10 fs	310 ± 20 fs		
	τ_2_	2.0 ± 0.2 ps	1.6 ± 0.1 ps		
	τ_3_	18 ± 5 ps	23 ± 5 ps		
	τ_4_	1.5 ns^b^	1.5 ns^b^		

a Error margins are statistical errors of the fits.

b Constrained to value determined by fitting of EOD data.

The stimulated emission evident in Figure 2a,b at wavelengths >600 nm was also observed by Ernst et al. and found to increase in intensity with probe wavelength. In the region between 540–700 nm it is overlapped by the positive ESA band. The 0.3 ± 0.1 ps time constant for decay of this stimulated emission reported by Ernst et al.^12^ is consistent with our measurements, and supports the proposed subpicosecond decay of the first bright state population.

The kinetics of the late time features are more challenging to extract from the TA spectra, given the weak signals, but suggest that the growth of the oNP^–^ anion occurs over time scales on the order of τ_4_ ≈ 1.5 ns with subsequent decay over τ_5_ ≈ 400 ns (Table 1 – fitting shown in SI Figure S6). Time constants of >100 ps, > 500 ps and 900 ± 50 ps, attributed to the decay of the triplet state, have been found in 2-propanol, *n*-hexane, and benzene, respectively.^12,16^ These values suggest that the triplet state could be sufficiently long-lived in aqueous solution to have a decay pathway to oNP^–^ via deprotonation with a time constant of ∼1.5 ns. Whether there is a competitive decay route in water allowing direct ISC from T_1_ back to S_0_ oNP is hard to determine from the data. In the TA spectra of aqueous solutions of the related nitrobenzene, T_1_ ESA bands are seen around 700 nm and <450 nm.^17^ Ernst et al. reported triplet ESA at around 480 nm in 2-propanol and *n*-hexane, but found negligible signal in water.^12^ Similarly, we are unable to attribute any features of the TA spectra to the triplet state.

Time-resolved infrared spectra shown in Figure 3 further support the band assignments in the TA spectra. Negative features in the TRIR spectrum of oNP in D_2_O, excited at 350 nm (Figure 3a–f) are ground state bleaches (GSBs) and correspond to the position of absorption peaks in the FTIR spectrum. A series of positive features (**a′**-**f′**), which emerge within the first few ps and shift to higher wavenumber with increasing time delay, are identified as HGSA peaks. Each is anharmonically shifted from an associated GSB feature **a**–**f** at slightly higher wavenumber and with matching decay kinetics. A positive feature in the late time (**A**) matches a major peak in the FTIR spectrum of the anion and is thus attributed to that species, in accord with our anion band assignment from TA spectra (band **iv** in Figure 2b). A second peak (**T**) which decays more quickly than **A**, is tentatively attributed to absorption from the oNP triplet state.

**Figure 3:**
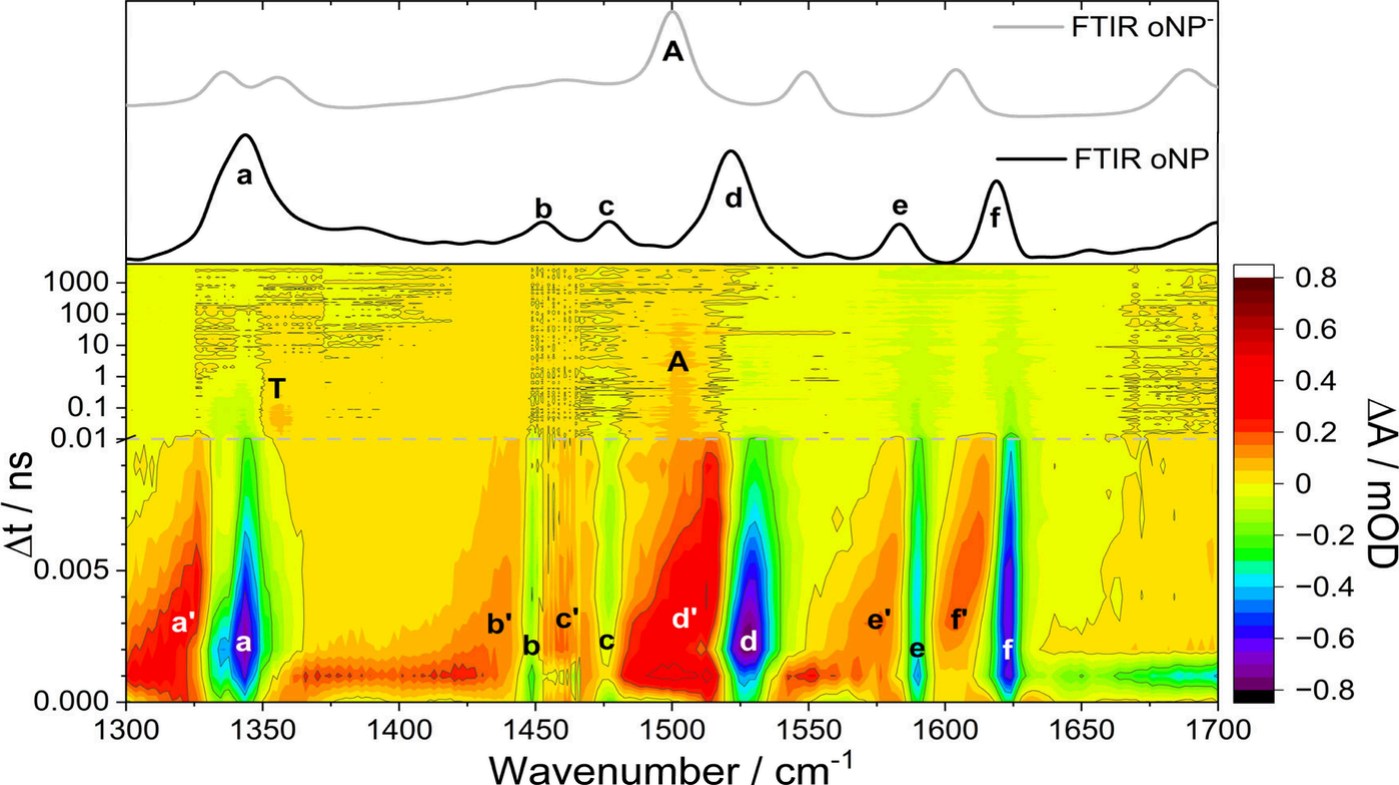
Time-resolved infrared spectra of a solution of oNP in D_2_O, pumped at 350 nm. Data are shown as a contour plot. The FTIR spectra of oNP and oNP^–^ in acidic and basic D_2_O respectively are provided for comparison. Features **a**–**f** are peaks in the FTIR spectrum of the neutral species and appear as ground state bleaches (GSBs) in the TRIR spectra; **a′**–**f′** are HGSA peaks located to the low wavenumber side of GSB features because of anharmonicity; **A** is a major peak in the anion FTIR which appears in the late time spectra. **T** is a weak absorption feature in the TRIR attributed to the triplet.

TRIR spectra also give an indication of the yields of the decay pathways.^17,18^ An exponential fit of integrated intensities for regions covering the two largest GSB and HGSA features in early time TRIR data gives decay time constants in the order of ∼3 ps (see SI section S4) which are comparable to the HGSA decay seen in the TA spectra. The majority of the GSB recovers within the first 20 ps, indicating that a route involving rapid decay of the first bright singlet state into S_0_ followed by vibrational relaxation is the dominant decay pathway. Fitting of the GSB recovery in the TRIR data suggests that this pathway accounts for 94% of excited species (see SI Figure S9 and Table S2). Quantum yields of the triplet state, and consequently the oNP^–^ anion, are therefore low. There is some evidence of bleach recovery in the range 100 ps −1 ns implying that there may be a pathway allowing direct ISC between T_1_ and S_0_ although this feature could also be due to overlapping absorption from the anion.

To better understand the competing relaxation pathways of oNP, LR-TDDFT within the Tamm-Dancoff approximation (TDA) and with the ωB97XD functional and aug-cc-pVDZ basis set was used to find critical geometries in the first excited electronic state. The outcomes are shown in Figure 4. The ground state minimum adopts the nitro form (Figure 1a). Excited state calculations found a minimum on the adiabatic S_1_ surface with nπ* character and a geometry representative of the S_1_/S_0_ intersection seam (IS) with ππ* character (Figure 4a). The intersection seam is accessed by excited state intramolecular proton transfer (ESIPT) from the OH group to the nitro group, forming the *aci* tautomer (Figure 1c), and torsion of the nitro group, as previously described by Ernst et al., among others.^12,15,19,20^ The computed geometry changes needed to access the intersection seam have recently been supported by gas-phase ultrafast electron diffraction data,^20^ and decay via this intersection has been shown to slow when rotation is inhibited by a more viscous solvent.^12^ The S_1_(nπ*) minimum is in the nitro form and corresponds to a reduction in the ∠ONO bond angle in the nitro group. This minimum has not been previously described because prior theory on oNP has concentrated on the region of the adiabatic S_1_ state with ππ* character.

**Figure 4:**
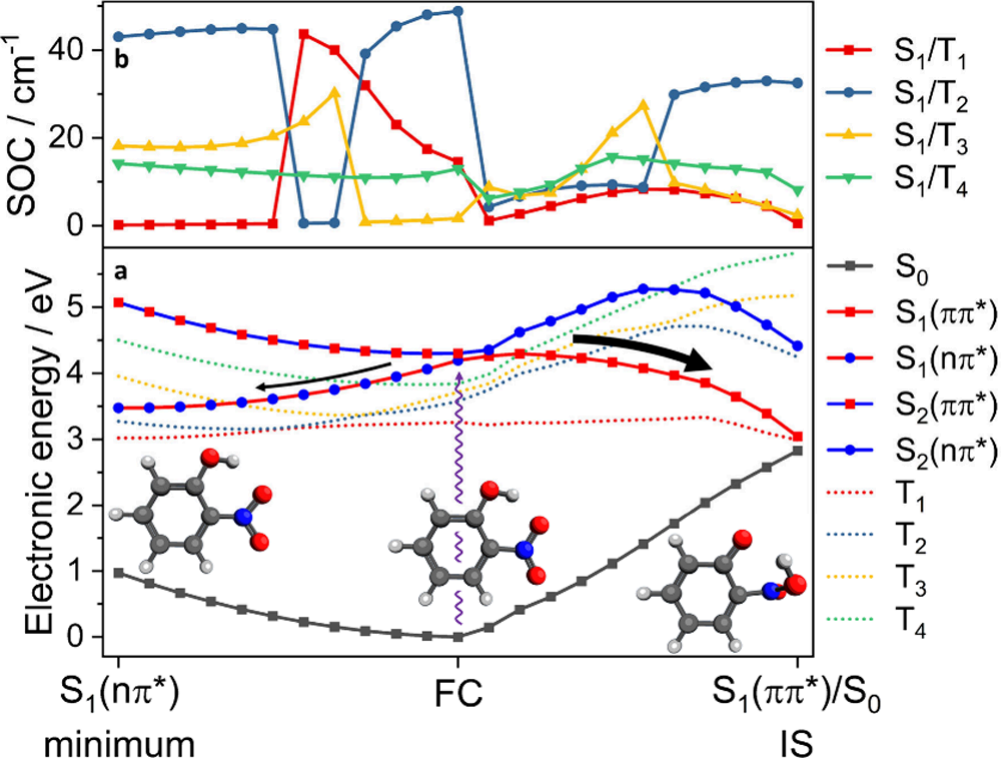
A linear interpolation in internal coordinates using 10 intermediate steps between geometries of the S_1_(nπ*) minimum, ground state minimum, and a geometry representative of the S_1_(ππ*)/S_0_ intersection seam. Energies of the ground and excited states relative to the S_0_ minimum (**a**) and the S_1_-T_n_ spin–orbit coupling (SOC) at each geometry (**b**) are calculated using (LR-TD)DFT/TDA/ωB97X-D3/ZORA-def2-TZVP. Continuous lines show the adiabatic singlet surfaces in **a**, point markers denote the character of the excited singlet states. A bold black arrow indicates the dominant relaxation pathway via the conical intersection, a thinner arrow the minor pathway proceeding to the S_1_(nπ*) minimum. All results shown are from gas-phase calculations.

The behavior of the potential energy surfaces was investigated along linear interpolation in internal coordinates (LIIC) pathways from the Franck–Condon (FC) geometry to the S_1_(ππ*)/S_0_ IS geometry (Figure 4a–rightwards) and to the S_1_(nπ*) minimum (Figure 4a–leftwards), with energies calculated using (LR-TD)DFT/TDA at the ωB97X-D3/ZORA-def2-TZVP level of theory in the gas phase. This level of theory was benchmarked with ADC(2) (see SI Figure S3). While care is needed when describing the S_1_/S_0_ intersection seam with LR-TDDFT,^21,22^ the pathway to the S_1_(ππ*)/S_0_ IS is consistent with findings using high-level wave function-based methods. The adiabatic S_1_ and S_2_ surfaces are of similar energy around the FC region, which can be interpreted as a crossing of the bright ππ* and dark nπ* diabatic surfaces. In this calculation, the first bright state is formally S_2_, but the ordering of the lowest bright and dark states is sensitive to the level of theory, basis set and solvation model used (see SI), hence why the first bright state is described as S_1_ in other work.^12,15,19^ Our calculated gas-phase LIICs suggest there is no energy barrier to either the S_1_(nπ*) minimum or the S_1_(ππ*)/S_0_ IS. A small energy barrier to the S_1_(ππ*)/S_0_ IS is apparent when solvation is modeled using a water CPCM (SI Figure S2). The influence of solvation on the potential energy surfaces is discussed below.

These computational data, alongside our TA and TRIR measurements, show that upon excitation at 350 nm into the first bright state, over 90% of molecules rapidly return to the ground state via a conical intersection (CI), accessed by proton transfer and NO_2_ torsion, with a time constant of ∼400 fs. These nonadiabatic dynamics produce vibrationally hot molecules in the electronic ground state which relax by vibrational energy transfer to the solvent with a time constant of ∼3 ps. The pathway proceeding from the FC region along the S_1_(ππ*) surface to the IS in Figure 4 is consistent with this major decay mechanism, but does not account for species entering the triplet manifold.

To investigate the possible formation of the triplet state of oNP, the spin–orbit coupling (SOC) magnitude between the first adiabatic singlet state S_1_ and the low-lying triplet states was calculated at each geometry as an indication of the probability of ISC (Figure 4b). Intersystem crossing from the region of the S_1_ surface of ππ* character is unlikely given the small SOC, larger singlet–triplet energy gaps, and the rapid decay of S_1_ population via the CI. A low likelihood of ISC from this surface was also identified by Nunes et al.^20^ A more plausible explanation is that ISC occurs from the nπ* region of the S_1_ surface. Significant SOC between S_1_ and T_2_ is calculated at the S_1_(nπ*) minimum where it has a magnitude of over 40 cm^–1^. T_2_ is of ππ* character at this geometry, and thus the significant SOC is in accordance with the El-Sayed rule.^23,24^ There is also a small energy gap of 0.2 eV between the two states at this geometry, indicating favorable conditions for ISC from internally excited S_1_ molecules. Favorable conditions for ISC from the S_1_(nπ*) state can also be explained by the fact that radiative decay back to S_0_ is symmetry forbidden, allowing time for ISC to occur. We cautiously attribute a weakly contributing signal observed in the TA spectra which decays with a time constant, τ_3_ ≈ 20 ps (Table 1), to absorption from the S_1_(nπ*) state which decays via this ISC. Assuming that ISC occurs primarily from geometries around this S_1_(nπ*) minimum, a bifurcation must first occur near the FC region on the ππ* state, leading to branching into nπ* and ππ* regions of the S_1_ surface.

Our experimental data show that, from the triplet state, oNP molecules deprotonate to form the anion–a process with a time constant in the order of 1.5 ns. This deprotonation must first make the anion in a triplet spin state, and ISC from T_1_ to S_0_ will contribute to the rate of formation of the oNP^–^ anion in its ground state. Reprotonation of the ground-state (S_0_) anion occurs on a time scale of ∼400 ns in aqueous solution at its intrinsic pH – but this time scale reduces significantly upon the addition of acid. In 0.1 M HCl, the reprotonation of the anion occurs on a time scale similar to, or faster than, its formation, which is why no features attributable to the anion are observable in the TA and TRIR data at pH 1.

Quantum chemical calculations help explain the proposed behavior of the triplet state. Following ISC into T_2_(ππ*) at geometries near the S_1_(nπ*) minimum, rapid internal conversion to T_1_(ππ*) is assumed to occur. A geometry optimization of this T_1_ state was performed using ωB97XD/aug-cc-pVDZ, first with LR-TDDFT/TDA and then using unrestricted DFT (UDFT) for the lowest energy triplet state. A LIIC from the FC region to the T_1_ minimum energy geometry was then performed using both LR-TDDFT/TDA and UDFT calculations (SI Figure S5). The resulting data suggest that the minimum energy geometry of the triplet state is the *aci* form (Figure 1c), as found by Ernst et al., among others,^7,12,25^ suggesting that ESIPT also occurs in the triplet state. Consequently, deprotonation from the triplet state will be from the protonated nitro group of the *aci* form, instead of the OH group found in the *nitro* form. Although calculating values for the SOC to the ground state by the chosen method is prone to error, the T_1_ - S_0_ SOC at the T_1_ minimum was found by LR-TDDFT/TDA (ωB97X-D3/ZORA-def2-TZVP) to be only 0.8 cm^–1^, accounting for the slow ISC to S_0_ that allows a competing decay pathway via deprotonation.

Considering the effects of the solvent, when calculations include implicit solvation by water using a CPCM, the nπ* states of oNP increase in energy due to stabilization of the electron density in nonbonding orbitals by solvent–solute interactions (SI Figures S1 and S2). The first bright state becomes formally S_1_, and access to the conical intersection therefore requires no change in the adiabatic surface. Implicit solvation by water moves the crossing of the lowest energy ππ* and nπ* diabatic surfaces further from the FC region, which is expected to result in lower branching onto the nπ* surface, and hence a lower triplet quantum yield. These effects are expected to become more pronounced in calculations that explicitly treat hydrogen bonding in a protic solvent.

From the TRIR data (Figure 3) it is evident that there is only a small quantum yield for the triplet state and thence the anion. This relaxation pathway is unfavorable because of low branching onto the nπ* surface in the FC region. The low quantum yield into the triplet state explains the absence of discernible contributions from this state to the TA spectra, and the low intensity of the oNP^–^ anion absorption band in the TA spectra, despite its relatively high molar absorption coefficient. The reported ESA from the triplet state of oNP photoexcited in other solvents may be indicative of a higher triplet quantum yield because of more favorable branching to the nπ* region of the S_1_ state. Recent work has shown how solvent effects can significantly influence the decay pathways of nitroaromatic compounds.^26^ The TRIR data also show a complete ground state bleach recovery on <1 μs time scales. This efficient regeneration of parent oNP in its S_0_ state is consistent with the very low quantum yields of phototransformation (5 × 10^–6^) reported previously for oNP in aqueous solution.^11^

The ultrafast photochemistry of near-UV excited oNP is dominated by an efficient relaxation pathway through a S_1_(ππ*)/S_0_ conical intersection accessed by intramolecular proton transfer and NO_2_ torsion which occurs on a 400 fs time scale, producing vibrationally excited molecules in the ground electronic state. Fewer than 10% of molecules instead enter the triplet manifold, most likely from the S_1_ state in a region of nπ* character following a branching near the FC region. Proton transfer to form the *aci* tautomer occurs on T_1_, after which decay of the triplet state is facilitated by deprotonation on the ns time scale. Aqueous oNP therefore has at least two nonradiative relaxation routes after excitation at 350 nm. These two pathways ensure a low quantum yield for photodegradation, making this species potentially environmentally persistent when dissolved in aqueous atmospheric aerosols.

## Experimental and Computational Methods

Ultrafast laser spectroscopy was conducted at the University of Bristol, and at the Central Laser Facility of the STFC Rutherford Appleton Laboratory using the LIFEtime instrument. Details of the laser setup at the University of Bristol are given in the SI section S6, whereas the LIFEtime setup is described elsewhere.^18,27^

For TA spectroscopy, aqueous solutions of oNP were prepared at an optical density of 0.4–0.5 at the pump wavelength and flowed continuously through cells with CaF_2_ windows with a 250-μm path length. Reservoirs of 100 cm^3^ were used, in case of photodegradation. Aqueous HCl was added to relevant samples at a concentration of 0.1 M. The pump pulse was set at 350 nm, the spot size and pulse energy at the sample to ∼150 μm fwhm and 0.5 μJ in the LIFEtime setup and ∼300 μm fwhm and 1 μJ in the setup at Bristol. In data collected at Bristol a white light continuum (WLC) was generated by focusing the 800 nm fundamental onto a CaF_2_ window. A long pass filter (λ > 360 nm) was used after the sample to remove pump scatter. For experimental measurements at LIFEtime, a WLC was generated by focusing the frequency doubled output of a Yb:KGW laser on sapphire to produce a continuum in the range 380–500 nm.^18^

For TRIR measurements, saturated solutions of oNP in D_2_O were prepared by sonication of an excess of the solid in D_2_O, followed by filtration. A small amount (∼10%) of excess D_2_O was added to prevent recrystallization. Concentrated DCl was added to relevant samples to create concentrations of 0.1 M. Solutions of volume ∼15 cm^3^ were flowed continuously through cells with CaF_2_ windows with a 100-μm path length. Fourier transform infrared (FTIR) spectra of oNP and its deprotonated anion were measured using near-saturated solutions held in similar cells with added DCl or NaOD, respectively. Pump characteristics were comparable with those used for TA at each facility. In data collected at Bristol a single IR probe pulse in the range 1450–1650 cm^–1^ was used. In data collected at LIFEtime, two synchronized overlapping IR probe pulses in the range 1280–1460 cm^–1^ and 1450–1700 cm^–1^ were used. All TA and TRIR data were processed and analyzed using KOALA software.^28^

Ground state DFT geometry optimization was conducted using the ωB97XD functional,^29^ and aug-cc-pVDZ basis set,^30,31^ using Gaussian 16 (RevA.03).^32^ LR-TDDFT geometry optimizations of excited states were conducted using the same functional and basis set. The Tamm-Dancoff approximation was used in all LR-TDDFT calculations.^33^ Frequency calculations were performed for all minima to confirm their nature. The aug-cc-pVDZ basis set was chosen to match that used by Ernst et al.,^12^ with ωB97XD chosen in place of B3LYP due to its inclusion of nonbonding interactions, and long-range correction.^29^ The geometry representative of the S_1_/S_0_ intersection seam was chosen as the geometry with the lowest S_1_ energy and closest S_1_/S_0_ approach (0.006 eV) in geometry optimization cycles of the S_1_(ππ*) state. Key geometries are provided as xyz files.

The geometries forming the linear interpolations in internal coordinates were obtained using Entos Envision.^34^ Ten intermediate geometries were used for the paths connecting S_0_ to S_1_(nπ*) or the S_1_(ππ*)/S_0_ IS. LR-TDDFT/TDA was used to calculate excitation energies, and singlet–triplet SOC,^35^ of the key and intermediate geometries using the ωB97X-D3 functional,^36^ and ZORA-def2-TZVP basis set,^37,38^ conducted in ORCA 5.0.3.^39,40^ The ZORA-def2-TZVP basis set was chosen as a suitable alternative to aug-cc-pVDZ using zeroth order regular approximation (ZORA) to the Dirac equation for scalar relativistic effects.^41^ ADC(2) calculations,^42^ conducted in ORCA using the aug-cc-pVDZ basis set, provided a comparison of the behavior of excited singlet states derived from a wave function-based method. Natural transition orbitals (NTOs)^43^ were calculated in ORCA. Visualization of surfaces and structures was performed in Avogadro,^44,45^ or IQMol.^46^

Unrestricted DFT calculations conducted in Gaussian were used to find minimum energy geometries of the lowest triplet state using ωB97XD/aug-cc-pVDZ. Unrestricted DFT, used to find the energy of the lowest triplet state, was performed in ORCA using ωB97X-D3/ZORA-def2-TZVP.

## Data Availability

Data are available at the University of Bristol data repository, data.bris, at https://doi.org/10.5523/bris.1z3j033v7744y2nax4q9q0w3r5.

## Supporting Information Available

Absorption band assignments, additional LIICs, NTOs, kinetic traces of transient absorption spectra, additional TRIR spectra, kinetics and fitting, investigation of isotope effects by TA spectroscopy, and a description of the laser setup at the University of Bristol (PDF)
Key geometries (ZIP)
