# Supplementary material for: Competing Nonadiabatic Relaxation Pathways for Near-UV Excited ortho-Nitrophenol in Aqueous Solution
Source: J Phys Chem Lett. 2024 Aug 29;15(36):9153–9. doi: 10.1021/acs.jpclett.4c02154 (PMC11403664; doi:10.1021/acs.jpclett.4c02154)
Supplement: Supplementary file 1 — jz4c02154_si_001.pdf [file jz4c02154_si_001.pdf]

Competing Non-Adiabatic Relaxation Pathways for Near-UV Excited *Ortho*-Nitrophenol in Aqueous Solution

Hallam J. M. Greene,[a] Deborin Ghosh,[a] Igor V. Sazanovich,[b] Ryan Phelps,[b] Basile F. E. Curchod,[a] and Andrew J. Orr-Ewing [a,*]

[a] School of Chemistry, University of Bristol, Cantock's Close, Bristol, BS8 1TS, UK

[b] Central Laser Facility, Research Complex at Harwell, Science and Technology Facilities Council, Rutherford Appleton Laboratory, Harwell Oxford, Didcot, Oxfordshire, OX11 0QX, UK

* Author for correspondence: a.orr-ewing@bristol.ac.uk

Table of Contents

S1. Further Computational Results S2

S2. Equations S6

S3. Further Transient Absorption Spectroscopy Data S7

S4. Further Time-resolved Infrared Spectroscopy Data S8

S5. Comparison of Kinetics in Water and D_2O - TA data S11

S6. Experimental - Description of Bristol Laser Setup S12

References S13

S1. Further Computational Results

Figure S1a shows transitions from the gas-phase ground state minimum, calculated at the ωB97X-D3/ZORA-def2-TZVP level of theory in the gas phase using ORCA.[1, 2] The two peaks in the UV/vis spectrum of aqueous oNP at 350 nm and 278 nm correspond to transitions labelled S_2 and S_4 respectively. LR-TDDFT also identifies two transitions with zero oscillator strength to states S_1 and S_3. There is an offset of around 0.7 eV between the theory and experiment, which can be partly explained by the absence of a description of solvation in the computed results, and partly by a tendency of the functional to overestimate excitation energies.[3]

Figure S1b-d also show transitions from the gas phase ground state minimum, calculated using LR-TDDFT at the ωB97X-D3/ZORA-def2-TZVP level of theory with a water CPCM (Figure S1b), and calculated by ADC(2)/aug-cc-pVDZ in the gas phase (Figure S1c) and with a water CPCM (Figure S1d). The ordering of the states S_1 and S_2 is particularly sensitive to solvation modelling. It should also be noted that when calculated by ωB97XD/aug-cc-pVDZ in the gas phase using Gaussian,[4] the bright $\pi\pi^*$ state is formally S_1 at the FC geometry. ADC(2) gives a better match to the experimental results, particularly when solvation is modelled. Figure S2 shows how the energies of excited singlet and triplet states change, along the LIIC calculated in Figure 3 in the main manuscript, when vertical transitions are calculated with solvation modelled by a water CPCM. The same key and intermediate geometries were used. The recalculated LIIC using a CPCM shows a small energy barrier along the LIIC towards the $S_1(\pi\pi^*)/S_0$ intersection seam (IS). There are two competing arguments for whether the associated excited-state intramolecular proton transfer (ESIPT) is a barrierless process in solution or not. On the one hand, inclusion of an explicit protic solvent may further increase the energy barrier along the LIIC. On the other hand, the given LIIC involves concerted proton transfer and NO_2 torsion through a series of intermediate geometries; there may exist a lower energy pathway deviating from the LIIC, such as a stepwise process, which may avoid this energy barrier.

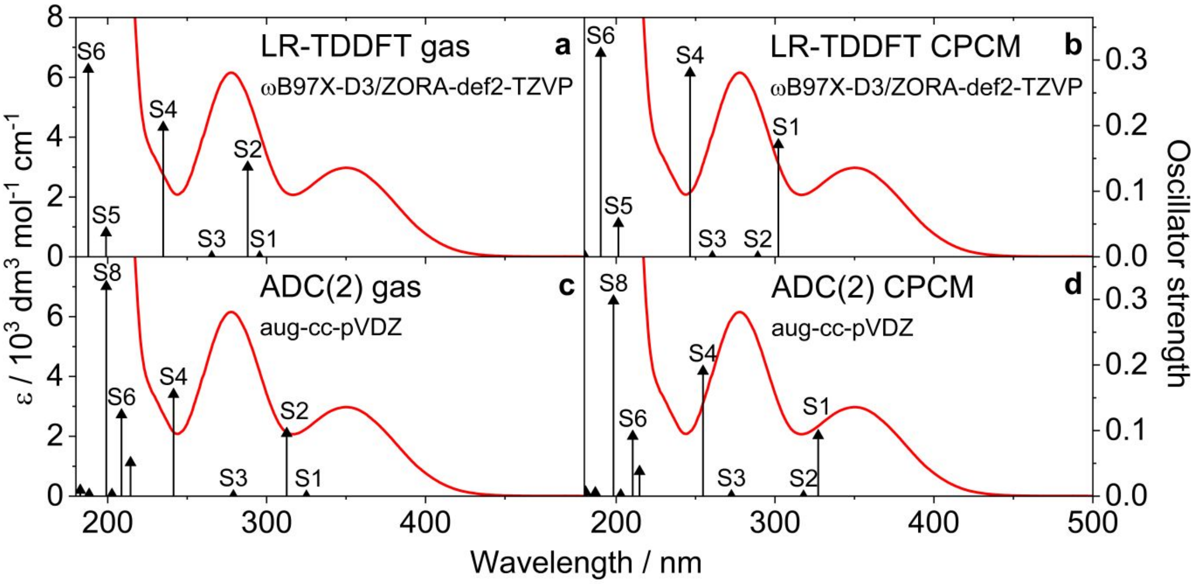

Figure S1. Experimental UV/vis absorption spectra of *ortho*-nitrophenol in aqueous solution (curves) with theoretical vertical excitations from the gas phase ground state minimum; calculated using LR-TDDFT/TDA/ωB97X-D3/ZORA-def2-TZVP in the gas phase (**a**) and with a water CPCM (**b**), and calculated using ADC(2)/aug-cc-pVDZ in the gas phase (**c**) and with a water CPCM (**d**).

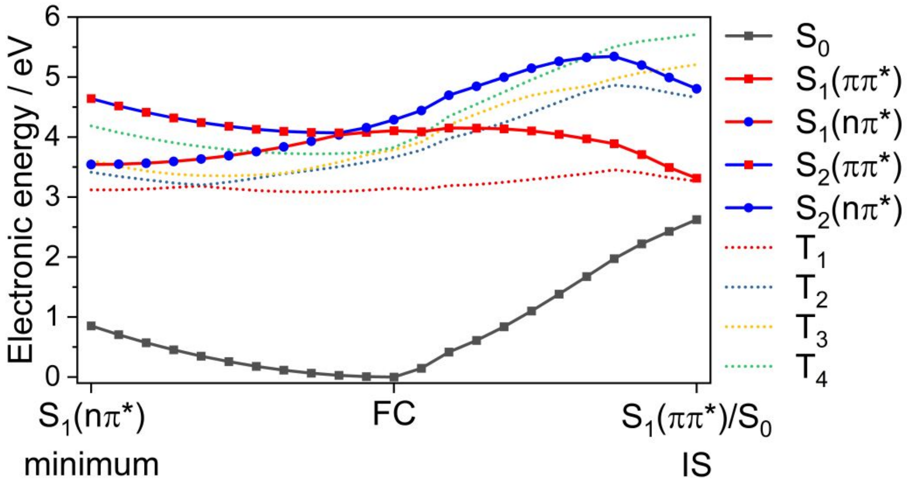

Figure S2. Electronic energies along the LIIC shown in Figure 3 of the main manuscript but using LR-TDDFT/TDA/ωB97X-D3/ZORA-def2-TZVP with a water CPCM and non-equilibrium solvation.

Figure S3 benchmarks the ωB97X-D3/ZORA-def2-TZVP calculations against ADC(2), showing similar trends in behaviour for the lowest 3 excited singlet states along the LIIC, although the excited states have higher energy when calculated by ωB97X-D3 compared to ADC(2).

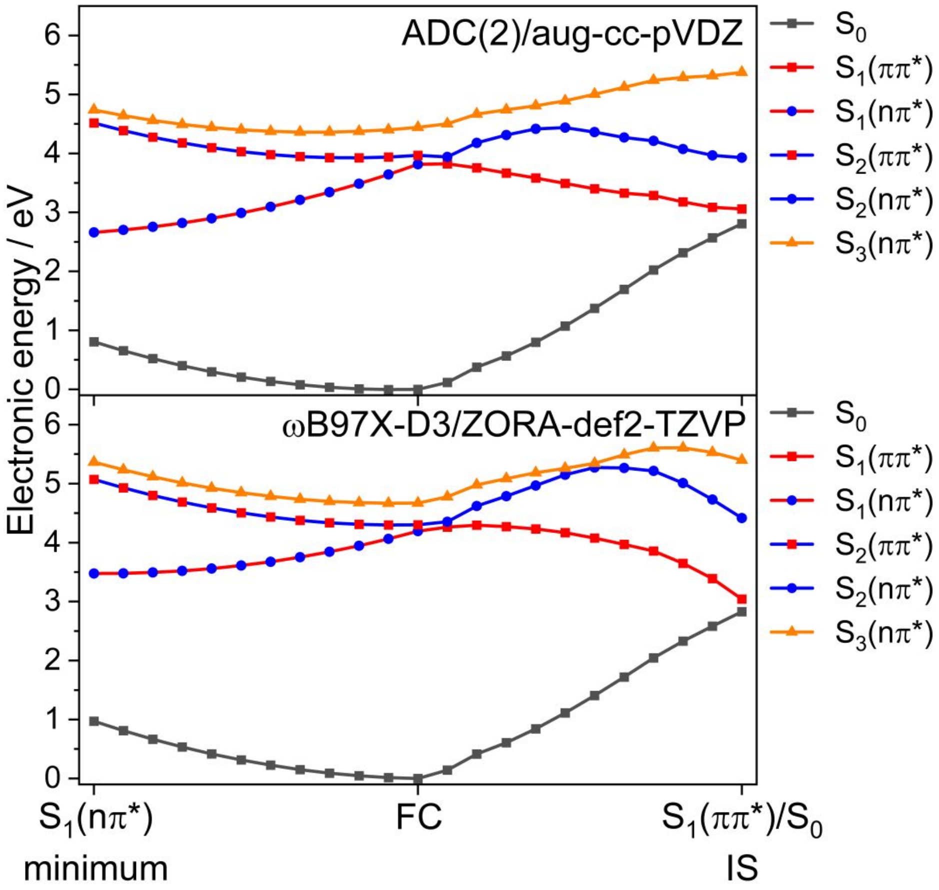

Figure S3. Comparison of the behaviour of the 3 lowest excited singlet states along the LIIC shown in Figure 3 of the main manuscript when calculated using ADC(2)/aug-cc-pVDZ and LR-TDDFT/TDA/ωB97X-D3/ZORA-def2-TZVP.

Figure S4 shows natural transition orbitals describing the character of S_1 and S_2 at key geometries. These allow the characterization of these states as nπ* or ππ*.

From the LIICs and NTOs (Figure S3 and Figure S4) it is also apparent that the S_2(nπ*) surface may itself consist of more than one diabatic surface. Near the FC region the non-bonding orbital is primarily located on the oxygen atoms of the nitro group, and nearer the intersection seam the non-bonding orbital has greater density on the oxygen of the phenol group. The change in energies of these diabatic surfaces along the LIIC can partly be explained by the proton transfer between the phenol and nitro groups.

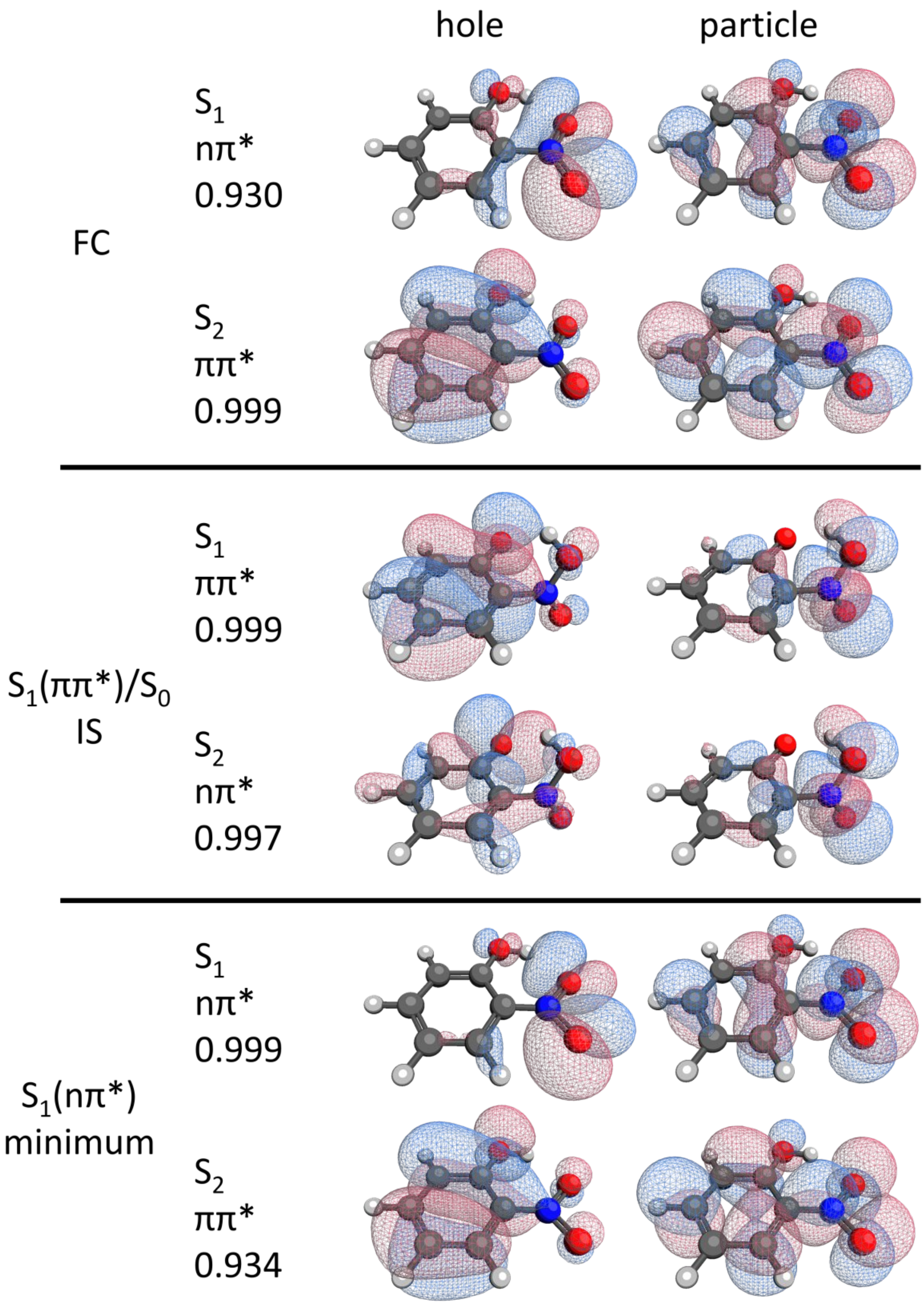

Figure S4. NTOs showing the character of the S_1 and S_2 excited singlet states, calculated by LR-TDDFT/TDA/ωB97X-D3/ZORA-def2-TZVP, at the geometries of the ground state minimum (FC – Franck-Condon), the geometry representative of the S_1(ππ*)/S_0 intersection seam, and the S_1(nπ*) minimum. Singular values for each pair of NTOs are reported.

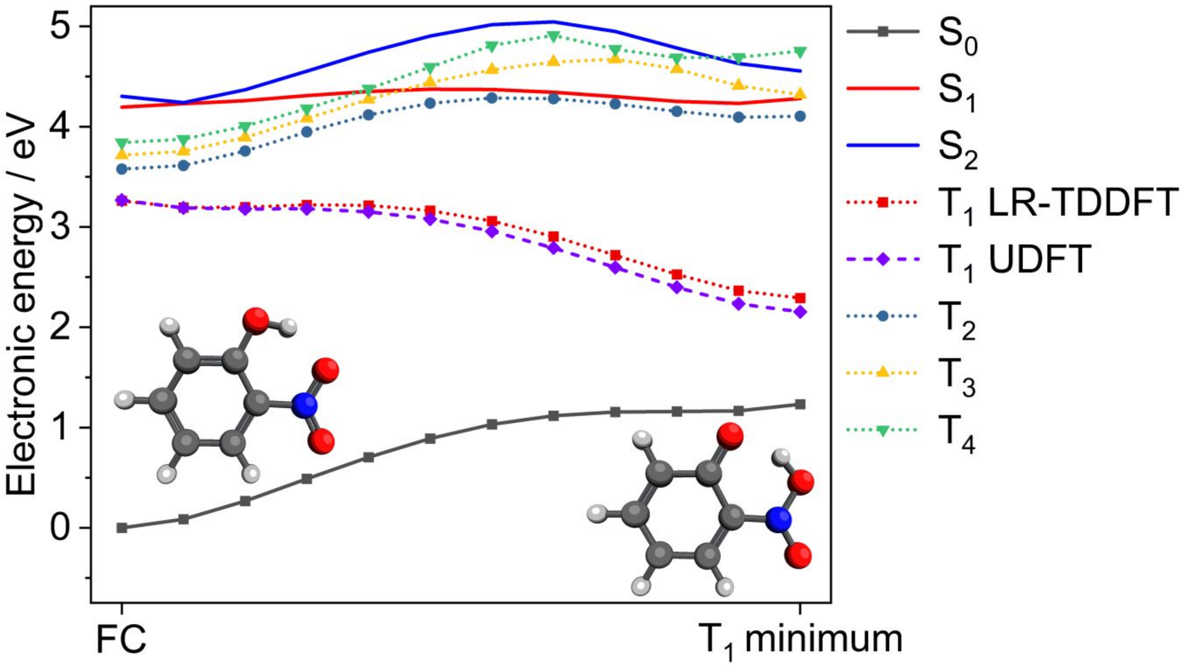

Figure S5. LIIC from the ground state minimum geometry to the T_1 minimum geometry with 10 intermediate steps with energies of the ground and excited states calculated at the (LR-TD)DFT/TDA/ωB97X-D3/ZORA-def2-TZVP level of theory, and the energy of the lowest triplet state calculated by unrestricted DFT/ωB97X-D3/ZORA-def2-TZVP.

S2. Equations

The SOC magnitudes reported in this work were calculated for each molecular structure using the following equation to account for all the triplet sublevels.

$$\left|H_{\mathrm{SOC}}^{IJ}\right| = \sqrt{\sum_{M_S=0,\pm1} \left|\left\langle \Phi_{\mathrm{singlet}}^{I} \middle| \hat{H}_{\mathrm{SOC}} \middle| \Phi_{\mathrm{triplet}}^{J,M_S} \right\rangle\right|^2}$$

S3. Further Transient Absorption Spectroscopy Data

Transient absorption spectroscopy data obtained over extended time delays using electro-optical delay methods (Figure S6) were used to determine the time constants of deprotonation of oNP to form oNP^{-}, and subsequent re-protonation. Time constants are give in Table 1 of the main manuscript.

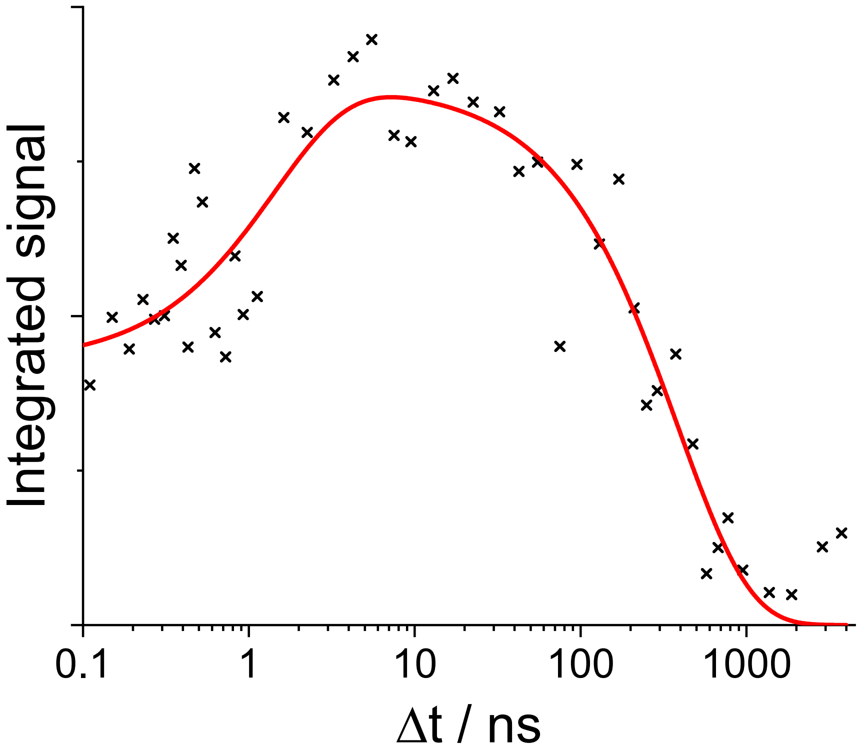

Figure S6. Kinetic traces for an integrated region from 400 – 475 nm from the transient spectra of oNP in water at its intrinsic pH, collected using electro-optic delays. Plotted points are the mean of pairs of adjacent experimental data points, and the red line shows a fitted biexponential curve.

S4. Further Time-resolved Infrared Spectroscopy Data

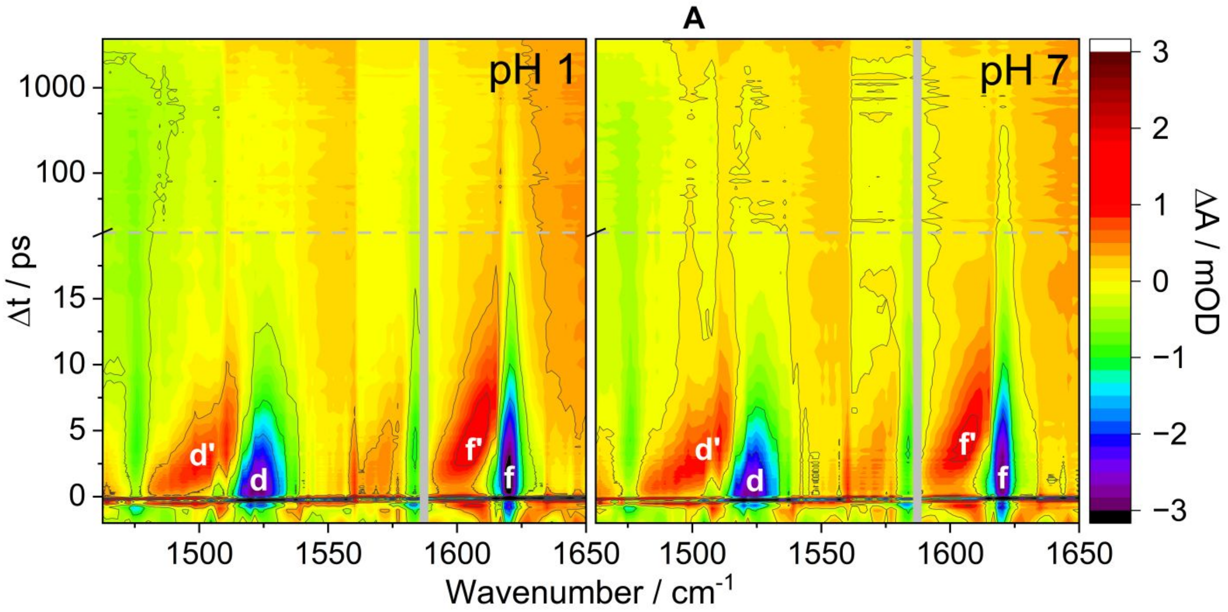

Figure S7. Contour plot showing the time-resolved infrared spectra of oNP in D_2O with 0.1 M DCl – denoted pH 1 – and without additional acid – denoted pH 7. GSBs **d** and **f** and corresponding HGSA **d'** and **f'** along with absorption attributed to the anion **A** are labelled similarly to Figure 2. The grey vertical bar is caused by a dead pixel in the detector.

TRIR spectra of aqueous (in D_2O) solutions of oNP obtained using only optical delays and a probe in the range 1450 cm^{-1} to 1650 cm^{-1} show the effect of added acid on the TRIR spectrum. There is evidence of the appearance of a peak at 1500 cm^{-1} (**A**) in the late time in the solution without added acid, which could be attributed to the anion. The spectra are otherwise alike.

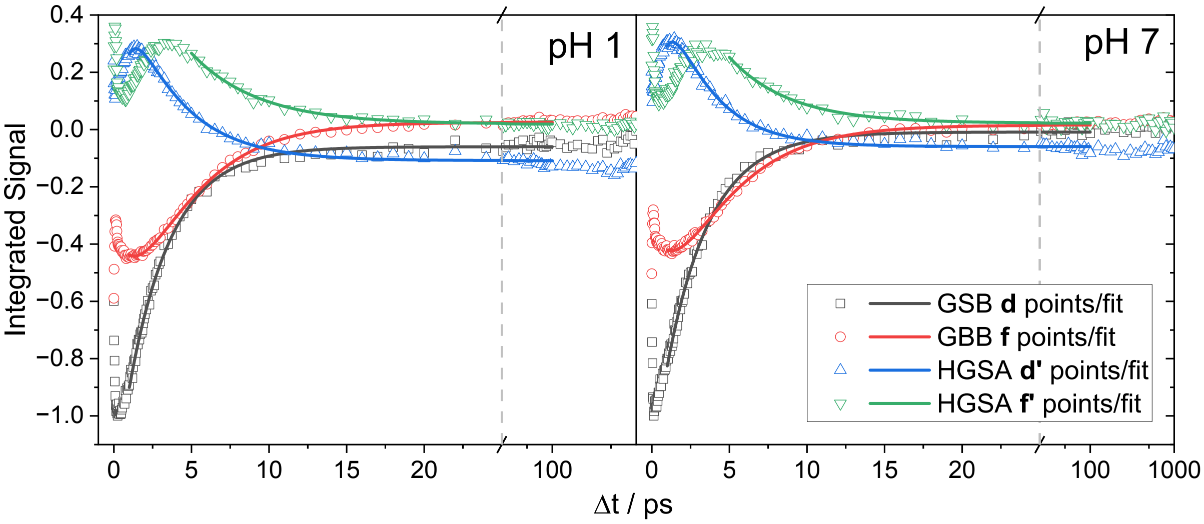

Figure S8. Kinetics of integration regions around bleaches **d** and **f** and HGSA features **d'** and **f'** of early time TRIR data gathered at Bristol (Figure S7). Bounds of integrals were determined by edges of positive or negative features at 100 fs, excepting **f'** where the dead pixel at ~1587 cm^{-1} was avoided. Data are fitted to bi- or mono-exponential decays, over the range 1- 100 ps, except for **f'**, which is fitted in the range 5 – 100 ps. Time constants are given in Table S1.

Table S1. Time constants derived from exponential fitting of integrated regions as shown in Figure S8. τ_1 represents a growth, τ_2 a decay. Error margins are statistical errors of the fits.

Feature	pH	τ_1 / ps	τ_2 / ps
GSB **d**	1	-	2.77 ± 0.03
	7	-	2.83 ± 0.04
GSB **f**	1	1.71 ± 0.37	3.52 ± 0.40
	7	1.70 ± 0.47	3.61 ± 0.48
HGSA **d'**	1	0.65 ± 0.12	3.44 ± 0.16
	7	0.67 ± 0.16	2.85 ± 0.20
HGSA **f'**	1	-	4.31 ± 0.25
	7	-	3.91 ± 0.29

A simple fit of integration regions covering the two largest bleach and HGSA features in the early time data (Figure S8, Table S1) gives time constants in the order of ~3 ps for the decay of both the GSB and HGSA features which are directly comparable to the HGSA decay seen in the TA spectra. The growth of HGSA **d'** is comparable to the 400-fs time constant seen in the TA and attributed to decay from the first bright state. Figure S8 also shows that the majority of the GSB recovers within the first 20 ps.

In the first 2-3 ps a feature overlaps GSB **f** and its corresponding HGSA **f'**. For GSB **f** this has been fitted with a time constant of 1.7 ps. Only the decay of **f'** has been fitted, once the contribution from this overlapping signal has reduced. The overlapping signal could be attributed to the bleach **e**, time zero artifact, ESA from the excited bright state, or distribution of vibrational energy across modes. The early time fall in the integrated signal of **f** and **f'** (Figure S8) suggests that, of these, overlap with the time zero artifact may be most likely.

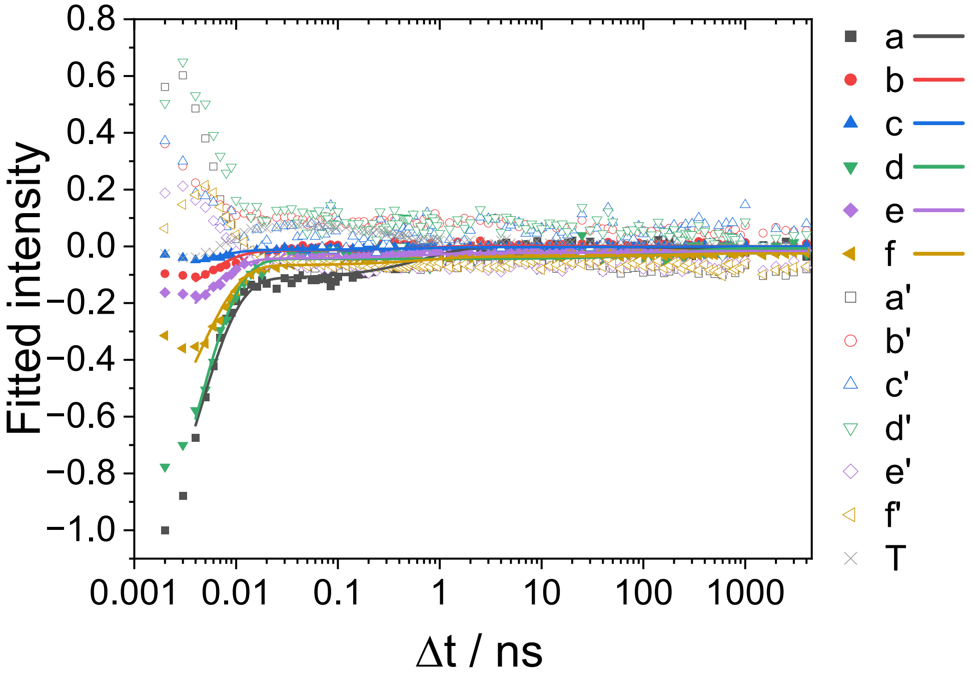

Figure S9. Kinetic traces extracted from the TRIR spectra of oNP in D_2O collected at RAL using electro-optic delays, fitted with Gaussian peaks. Kinetic traces are fitted to a triexponential function in the range 4 ps – 4 µs.

Table S2. Time constants and amplitudes from the triexponential global fitting of data shown in Figure S9.

Bleach	a	b	c	d	e	f
y_0	-0.008 ± 0.002	0 ± 0.003	0 ± 0.003	-0.007 ± 0.003	-0.017 ± 0.002	-0.025 ± 0.003
A_2	-1.41 ± 0.05	-0.29 ± 0.02	-0.12 ± 0.02	-1.54 ± 0.05	-0.42 ± 0.03	-0.93 ± 0.03
τ_2			0.0040 ± 0.0001 ns			
A_4	-0.114 ± 0.004	-0.017 ± 0.004	-0.010 ± 0.004	0 ± 0.004	-0.020 ± 0.004	-0.031 ± 0.004
τ_4			0.73 ± 0.06 ns			
A_5	0.008 ± 0.004	0.001 ± 0.004	-0.001 ± 0.004	-0.037 ± 0.004	-0.006 ± 0.004	-0.011 ± 0.004
τ_5			220 ± 70 ns			
Reduced χ^2			1.41894×10^{-4}			

By comparing the relative magnitudes of A_x it was possible to estimate the fraction of ground-state recovery proceeding via the $S_1(\pi\pi^*)/S_0$ conical intersection. Time constants for oNP in D_2O differ slightly from those in water, which may be because of a kinetic isotope effect (see Section S5).

S5. Comparison of Kinetics in Water and D_2O - TA data

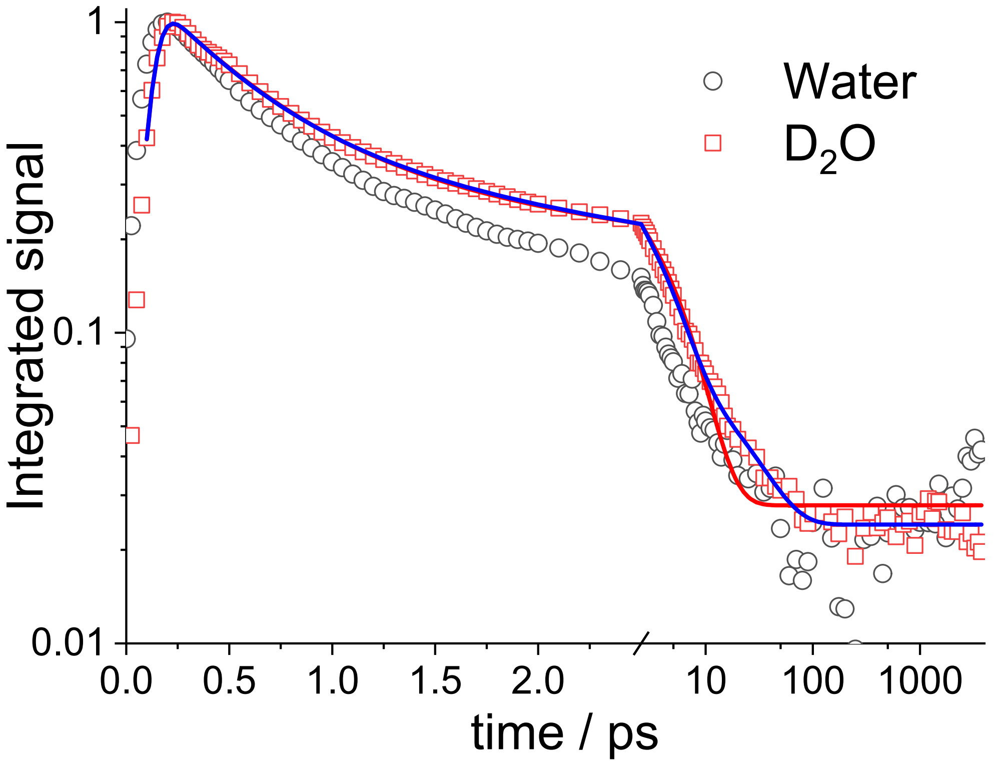

Figure S10. Kinetic traces for a wavelength-integrated region from 400 – 600 nm, from the transient absorption spectra of oNP in D_2O and water, in both cases at the intrinsic pH (pD) of oNP. The data from the solution in D_2O are fitted to a modified biexponential function (red) or higher-order exponential (blue).

Table S3. Time constants derived from wavelength-integrated regions of the TA data. Error margins are statistical errors of the fits.

	Dataset	D_2O
Biexponential fitting	Time window fitted	0.1 – 1000 ps
	τ_1	490 ± 10 fs
	τ_2	4.7 ± 0.2 ps
Higher order fitting[a]	Time window fitted	0.1 – 3750 ps
	τ_1	450 ± 10 fs
	τ_2	3.1 ± 0.2 ps
	τ_3	24 ± 5 ps

[a] In the absence of measurements at longer time delays, no attempt was made to fit the growth of the anion peak.

TA data for oNP in D_2O indicate that the time constants for all processes differ slightly from those in water. The difference in τ_1 is subtle, but the larger value in D_2O could indicate a kinetic isotope effect, which may benefit from further study. The slower cooling of the hot ground state in D_2O can also be explained by the different density of vibrational states of the solvent. Other time constants involving the breaking or formation of O-H/O-D bonds, which occur too slowly to be fitted from this data, will also be affected by the solvent isotope effect.

S6. Experimental - Description of Bristol Laser Setup

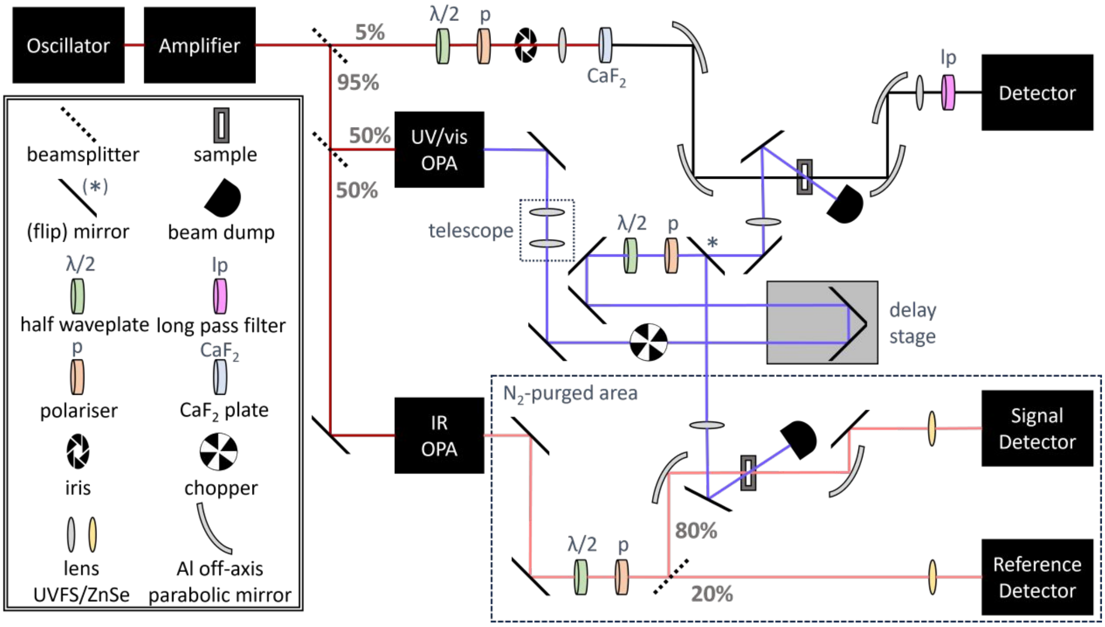

Figure S11. Simplified schematic diagram of the transient absorption (TA) spectroscopy and time-resolved infrared (TRIR) spectroscopy setups at the University of Bristol.

For time-resolved infrared (TRIR) data collected at the University of Bristol, using the apparatus shown schematically in Figure S11, the output of a Ti:sapphire laser (Coherent Astrella - 1 kHz, 800 nm, 7 W, 35 fs pulse width) was split equally between two optical parametric amplifiers (OPAs – Coherent OPerA Solo) generating tuneable pulses in the UV/visible and mid-infrared. The UV/visible pulse, used as a pump, was collimated, and passed through a 500 Hz mechanical chopper to allow sequential pump-on, pump-off measurements. The pump was aligned through a 60-cm delay stage allowing optical delays of up to ~4 ns. The tuneable broadband mid-IR output from the OPA was used as a probe, with 20% delivered to a reference 128-element MCT (Mercury Cadmium Telluride) detector mounted in an IR spectrometer. The remaining 80% was overlapped with the optical pump and focussed at the sample position, before being focussed into a matching MCT detector mounted in a separate IR spectrometer.

For TA measurements, a white light continuum (WLC) probe in the range 300-750 nm was generated by focusing a small portion of the 800-nm fundamental onto a CaF_2 plate. The pump and probe were focussed and overlapped at the sample position, and the probe subsequently focussed into a detector after passing through a low pass filter to remove pump scatter.

In both TA and TRIR measurements, the pump and probe were linearly polarised at the magic-angle geometry. The probe was focussed to a spot approximately half the area of the pump spot. The TA IRF was fitted with a FWHM of ~120 fs.

References

(1) Neese, F. The ORCA program system. *Wiley Interdiscip. Rev. Comput. Mol. Sci.* **2012**, *2* (1), 73-78. DOI: 10.1002/wcms.81.

(2) Neese, F. Software update: The ORCA program system—Version 5.0. *Wiley Interdiscip. Rev. Comput. Mol. Sci.* **2022**, *12* (5), e1606. DOI: 10.1002/wcms.1606.

(3) Bhatta, R. S.; Pellicane, G.; Tsige, M. Tuning range-separated DFT functionals for accurate orbital energy modeling of conjugated molecules. *Comput. Theor. Chem.* **2015**, *1070*, 14-20. DOI: 10.1016/j.comptc.2015.07.022.

(4) Frisch, M. J.; Trucks, G. W.; Schlegel, H. B.; Scuseria, G. E.; Robb, M. A.; Cheeseman, J. R.; Scalmani, G.; Barone, V.; Petersson, G. A.; Nakatsuji, H.; Li, X.; Caricato, M.; Marenich, A. V.; Bloino, J.; Janesko, B. G.; Gomperts, R.; Mennucci, B.; Hratchian, H. P.; Ortiz, J. V.; Izmaylov, A. F.; Sonnenberg, J. L.; Williams; Ding, F.; Lipparini, F.; Egidi, F.; Goings, J.; Peng, B.; Petrone, A.; Henderson, T.; Ranasinghe, D.; Zakrzewski, V. G.; Gao, J.; Rega, N.; Zheng, G.; Liang, W.; Hada, M.; Ehara, M.; Toyota, K.; Fukuda, R.; Hasegawa, J.; Ishida, M.; Nakajima, T.; Honda, Y.; Kitao, O.; Nakai, H.; Vreven, T.; Throssell, K.; Montgomery Jr., J. A.; Peralta, J. E.; Ogliaro, F.; Bearpark, M. J.; Heyd, J. J.; Brothers, E. N.; Kudin, K. N.; Staroverov, V. N.; Keith, T. A.; Kobayashi, R.; Normand, J.; Raghavachari, K.; Rendell, A. P.; Burant, J. C.; Iyengar, S. S.; Tomasi, J.; Cossi, M.; Millam, J. M.; Klene, M.; Adamo, C.; Cammi, R.; Ochterski, J. W.; Martin, R. L.; Morokuma, K.; Farkas, O.; Foresman, J. B.; Fox, D. J. *Gaussian 16 (Rev. A.03)*; Wallingford, CT, 2016.
